# Tin–Lead Liquid Metal Alloy Source for Focused Ion Beams

**DOI:** 10.3390/mi17010076

**Published:** 2026-01-06

**Authors:** Bryan Flores, Shei Sia Su, Coleman Cariker, Ricardo A. Dacosta, Aaron M. Katzenmeyer, Alex A. Belianinov, Michael Titze

**Affiliations:** 1 Sandia National Laboratories, Albuquerque, NM 87123, USA; bflor058@ucr.edu (B.F.); cbcarik@sandia.gov (C.C.); rdaco002@ucr.edu (R.A.D.); aabelia@sandia.gov (A.A.B.); 2 Department of Material Science & Engineering, Bourns College of Engineering, University of California, Riverside, CA 92521, USA; 3 National Institute of Standards and Technology, Gaithersburg, MD 20899, USA; 4 Luxembourg Institute of Science & Technology, L-4422 Belvaux, Luxembourg

**Keywords:** focused ion beam, liquid metal alloy, ion source, eutectic, color center

## Abstract

Focused Ion Beam (FIB) systems are increasingly utilized in nanotechnology for nanostructuring, surface modification, doping, and rapid prototyping. Recently, their potential for quantum applications has been explored, leveraging FIB’s direct-write capabilities for in situ single ion implantation, which is crucial for fabricating single photon emitters. Color centers in diamond can function as qubits and are of particular interest due to their capacity to store and transmit quantum information. While Group-IV color centers exhibit high brightness, they require low temperatures to retain coherence. However, lead-vacancy in diamond (PbV) operates at the higher end (4 K) of this temperature spectrum due to larger ground-state splitting, making them particularly interesting. In this context, our study presents results for lead (Pb)-containing alloys with eutectic points below 600 °C and results on using tantalum (Ta) and titanium (Ti) as emitter materials for a Pb liquid metal alloy ion source. We show that a standard FIB system is able to resolve the different Pb isotopes and achieve nanoscale spot sizes, as required for quantum information science applications.

## 1. Introduction

**Focused** Ion Beam (FIB) systems are widespread in nanotechnology for nanostructuring, local surface modification, doping, and rapid prototyping [1,2]. Recently, FIBs have also been used for quantum applications [3,4,5]. Here, FIBs offer a unique advantage by being a direct-write technique, enabling in situ process control and allowing for single ion implantation for the fabrication of single photon emitters [6,7,8]. For quantum defects, the ability to implant a variety of ions with high spatial and mass resolution is critical for (i) creating single photon emitters within the highest field region of optical cavities [9,10] and (ii) selecting specific isotopes to enable control over hyperfine splitting in the implanted color centers [11,12], which can be used for long-term information storage [13,14,15].

Color centers in diamond can act as qubits, as they can store and transmit quantum information [16,17]. Group-IV-based color centers in diamond have high brightness but require low temperatures for operation [18,19]. This low temperature operation requirement of Group-IV color centers is rooted in the strength of ground-state splitting, leading to a loss of coherence at elevated temperatures. The ground-state splitting of Sn and Pb, however, is high enough to enable operation at 1.8 K and >4 K, respectively, making them exceptionally interesting color center candidates [20,21]. While Pb-containing FIB sources have previously been reported in the literature, their reported melting points exceed 240 °C [22,23], leading to a larger source energy spread than eutectic Sn_74_Pb_26_ (at.%) with a melting point of 182 °C [24].

Herein, we present results on an exhaustive search of the ASM Alloy phase diagram database, identifying Sn_74_Pb_26_ (at.%) as a viable liquid metal alloy ion source (LMAIS) material [25]. We use Ta and Ti as the emitter materials to enable a Pb LMAIS. The Sn_74_Pb_26_ (at.%) eutectic is an ideal candidate material for an FIB LMAIS due to its low melting point [24], low source energy spread [26], and implantation of either Sn or Pb from a single source. However, while tungsten emitters are widely commercially available, eutectic Sn_74_Pb_26_ (at.%) alloy wets the tungsten poorly, and thus the LMAIS does not emit effectively. We report Ta and Ti metals as an alternative to the emitter material for improved wettability. Our results show for the first time isotopically resolved Pb in an FIB.

## 2. Source Selection

**Figure 1:**
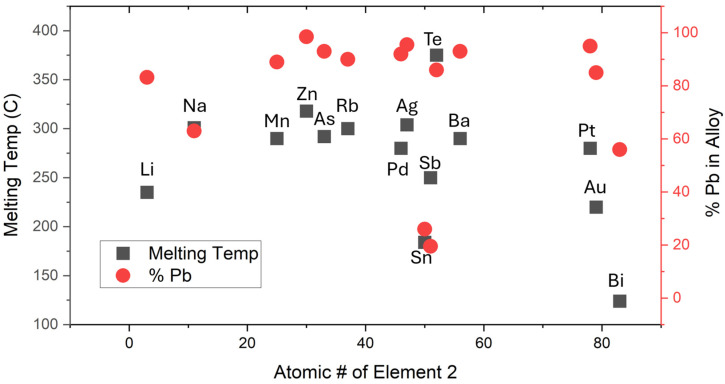
Results from mining the ASM International phase diagram database for binary eutectic and eutectic-like alloys that contain Pb and have a eutectic phase transition below 600 C. The x-axis is the atomic number of the other element in the alloy.

Binary alloy phase diagrams were automatically evaluated for the presence of a eutectic alloy and, if a eutectic is formed, to determine the eutectic composition and melting point. This was accomplished using ASM International’s Materials Platform for Data Science API access, through which machine-readable binary phase diagrams are accessible. Eutectic-like points are also included in our search and identified as points on the liquidus line where the derivative is discontinuous at a local minimum. For eutectic and eutectic-like alloys, the composition and melting point are evaluated. If an alloy contains multiple eutectic-like points, the one with the lowest phase transition temperature is used. Even in the case of peritectic and complex eutectic reactions, these phase diagram qualities identify a binary alloy as prospectively good for use as an LMAIS. This distilled product taken from the ASM database therefore provides a useful tool for quickly identifying LMAIS candidates from known and characterized binary alloys. Here, the results were filtered to include only alloys that contain Pb and have eutectic transitions below 600 °C and are shown in Figure 1. Limitations on the available heating current in the nI, as well as excessive chromatic aberrations, require alloys with a melting point below 600 °C. The x-axis of Figure 1 shows the atomic number of the second element in the binary eutectic. The black squares correspond to the left y-axis, which denotes the melting point of the alloy at the eutectic composition. The red circles correspond to the right y-axis, which denotes the Pb content at the eutectic point. Sn_74_Pb_26_ (at.%), a well-known eutectic used in soldering, is found to have the second lowest melting point of all Pb-containing binary alloys and is therefore used for fabrication of an LMAIS. The coexistence of Sn and Pb in a single source makes this an attractive source for the fabrication of diamond color centers, as both SnV and PbV are attracting significant interest due to their relatively high operating temperature, avoiding the need for dilution refrigerators. Overall, source selection for Sn_74_Pb_26_ (at.%) was made due to the low melting point, availability, and low cost of the alloy and the wettability of the Ti and Ta wires. Potential future work could explore the use of Pb_45_Bi_55_ (at.%) with an even lower melting point than Sn_74_Pb_26_ (at.%), given sufficient wettability of emitter materials.

## 3. Experimental Methods

This work utilizes two experimental setups: (1) a source preparation unit (SPU), in which filaments can be filled with alloys and emission testing can be performed, and (2) the A&D FIB 100 nI nanoImplanter (A&D Co. Ltd., Tokyo, Japan) hereafter denoted as “nI”, a 100 kV Focused Ion Beam system with a Wien filter for separating different elements from an LMAIS, focusing optics, and a Faraday cup on the sample holder. Imaging the nI is performed using secondary electron detection. This machine has previously been described elsewhere [27].

## 4. Results

**Figure 2:**
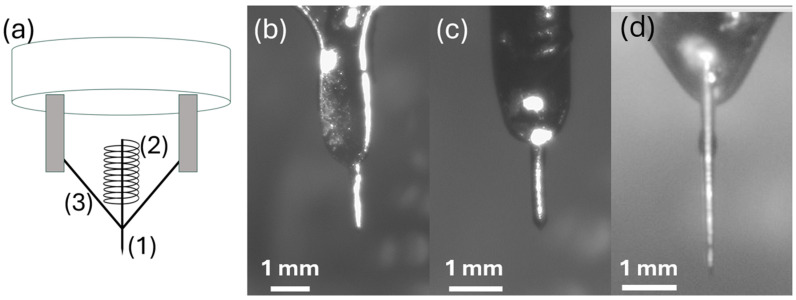
Source geometry. (**a**) A schematic highlighting the overall emitter geometry and relevant features. (**b**) Ti-based emitter source after filling. (**c**) Ta-based emitter source after filling. The scale bar in both images denotes 1 mm. (**d**) W-based emitter showing incomplete wetting of the needle.

Figure 2a shows a schematic of the source geometry, highlighting the three most relevant features: (1) the tip, which is formed by grinding the wire to a point to enhance emitter sharpness and field concentration; (2) a reservoir wound from the wire; and (3) a piece of wire used for conducting the current to enable Joule heating of the emitter. Figure 2b shows the Ti-based emitter, and Figure 2c shows the Ta-based emitter after successful wetting with Sn_74_Pb_26_ (at.%). In both cases, the reservoir is filled and the needle is covered with the Sn_74_Pb_26_ (at.%) eutectic. While we cannot measure the temperature of the emitter during operation, the emitter does not glow during operation, indicating an operation temperature significantly below the temperature of our AuSi-based emitters with a melting point of 360 °C. We show an incompletely wetted commercial W emitter in Figure 2d. In previous work, we have used scanning electron microscope-based energy dispersive spectroscopy (EDS) to elucidate the composition of the material after tip fabrication, showing oxidation of reactive alloy materials [28]. Here, the alloy maintains its metallic shine after melting, and the same alloy was used to fill the Ti and Ta emitters, indicating that the lack of wetting is not due to an oxide forming on the alloy surface. The emitters are made from bare 0.25 mm diameter wires of Ti and Ta. While these emitters were made to resemble the geometry of commercial tungsten emitters, our emitter tips were not electrochemically etched.

**Figure 3:**
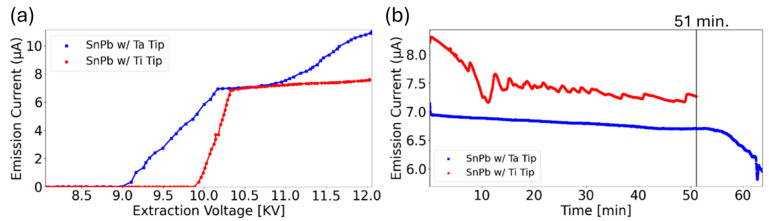
IV performance of Sn_74_Pb_26_ (at.%) sources. (**a**) IV curves of the two emitters. (**b**) Stability of the Sn_74_Pb_26_ (at.%) source at 10 kV extraction voltage. For both graphs, the blue curve is for the source on the Ta emitter, and the red curve denotes the source on the Ti emitter.

We tested our emitters multiple times to verify the emitters’ current-voltage (IV) characteristics and stability. The IV curves in Figure 3a show that both LMAIS sources have a turn-on voltage below which field ionization does not occur. The Ta-based emitter turns on at 9.18 kV, while the Ti-based emitter turns on at 9.88 kV. The turn-on is followed by a regime where the current increases linearly with increasing extractor voltage. While the Ta-based emitter in this regime has a slope of 6 × 10^−6^ A/kV, the Ti-based emitter has a much steeper slope of 2 × 10^−5^ A/kV. For both emitters, the linear regime is followed by a saturation regime starting at 10.17 kV for the Ta-based emitter and 10.32 kV for the Ti-based emitter. The differences in the turn-on and IV behaviors are ascribed to small differences in sizes and shapes of the emitters inherent to the handmade fabrication process, rather than differences in material performance. We also note an important detail regarding the emitter stability tests: the IV curves show how the LMAIS behave when the extraction voltage is running in different regimes. The stability tests were performed with the extraction voltage set at 10 kV for both emitters, which is in the linear regime for the Ti-based emitter where the emission current changes significantly for small changes in the extraction voltage. In contrast, the 10 kV setting for the Ta-based emitter is in the saturation regime, where the IV is mostly flat leading to smaller changes in emission current with extraction voltage fluctuations.

In the stability test shown in Figure 3b, both sources were operating at an extraction voltage of V_ext_ = 10 kV. Both sources show a slow downward trend in emission current, likely due to insufficient heating of the source material, as the emitters are operated without any heat shielding such that radiative cooling is significant. Our previous work suggests that sources with stability, as shown in Figure 3b, perform better in the FIB system, likely due to the proximity to the suppressor and extractor electrodes acting as heat shields, leading to better temperature stability [29].

The Ti-based LMAIS is more unstable throughout the run due to it operating in the linear regime. In the saturation regime, we expect more stable operation for the Ti-based source. In contrast, the Ta-based source is run in the saturation regime, exhibiting good stability for ~ 1 h. Overall, we find that source performance is strongly dependent on the specific emitter but IV behavior is in line with what is required for an FIB source. The stability is sufficient for use in FIB applications, which we show below.

**Figure 4:**
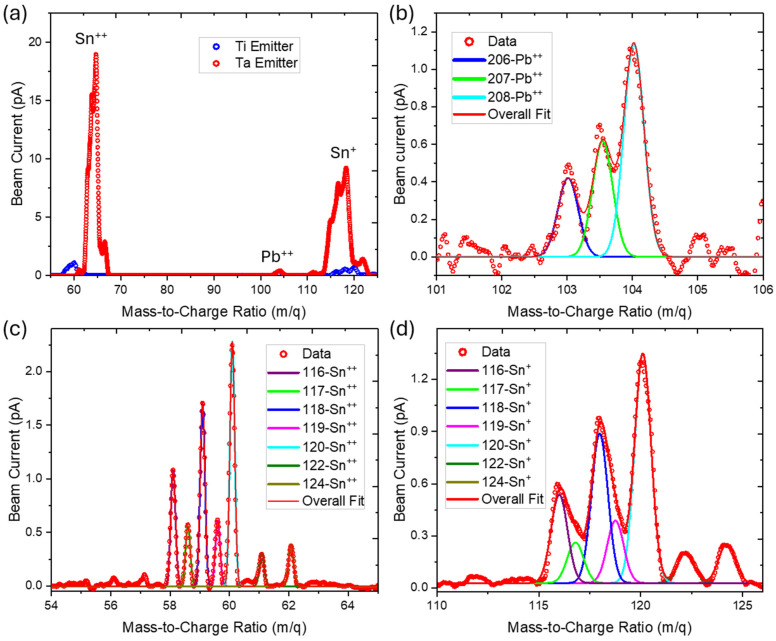
Mass spectra of Sn_74_Pb_26_ (at.%) sources. (**a**) Overall mass spectrum showing Sn^++^, Pb^++^, and Sn^+^ peaks fabricated on Ti (blue) and Ta (red) emitter. (**b**) Zoomed-in view of Pb^++^ part of the mass spectrum. Each isotope is fit to a Gaussian shown as the blue (206-Pb), green (207-Pb), and cyan (208-Pb) lines. The overall fit is shown as the red line. (**c**) Zoomed-in view of Sn^++^ part of the mass spectrum. Each isotope is fit to a Gaussian with the overall fit shown as the red line. (**d**) Zoomed-in view of Sn^+^ part of the mass spectrum. Each isotope is fit to a Gaussian with the overall fit shown as the red line.

Figure 4 shows the mass spectra in terms of mass-to-charge ratios collected at 80 kV acceleration potential. The electric field of the Wien filter was set to 1.625 kV/cm, and the magnetic field was swept to acquire the mass spectrum. Figure 4a shows a full mass spectrum sweep of both Ti-based and Ta-based emitters. We expect that single charged Pb and larger clusters are also emitted from the source; however, overheating of the electromagnet prevented us from going to higher magnetic fields to observe these charge states. Figure 4b–d show high-resolution mass spectra of the Pb^++^, Sn^++^, and Sn^+^ peaks for the Ta-based emitter. A different SnPb source with approximately 12.8–14% Pb content has previously been reported in [22]. This source composition deviates from the eutectic composition utilized in this work, requiring a higher temperature to fully melt the source material. Fitting the isotopically resolved peaks for Sn^2+^, Sn^1+^, and Pb^2+^, we obtain the mass resolution for the QIS-relevant nuclear spin ½ isotopes: m/Δm = 333 for 207-Pb^++^, 447 for 117-Sn^++^, and 144 for 117-Sn^+^. To perform the fitting and ensure realistic mass resolutions, the width of the peaks within each ion species is shared between all peaks. This avoids underestimation of the mass resolution of the less abundant isotopes. The mass resolution achieved here for 117-Sn^++^ is comparable to that published in previous work [30]. From the fitting to the mass spectra, we calculate relative ionization ratios. These are found to be Sn^+^/Sn^++^ = 5.25 and Pb^++^/Sn^++^ = 1.86, respectively.

The absence of Ta and Ti in the mass spectra shows that the emitter materials do not chemically react with the alloy, as they would have been observed in the mass spectra.

**Figure 5:**
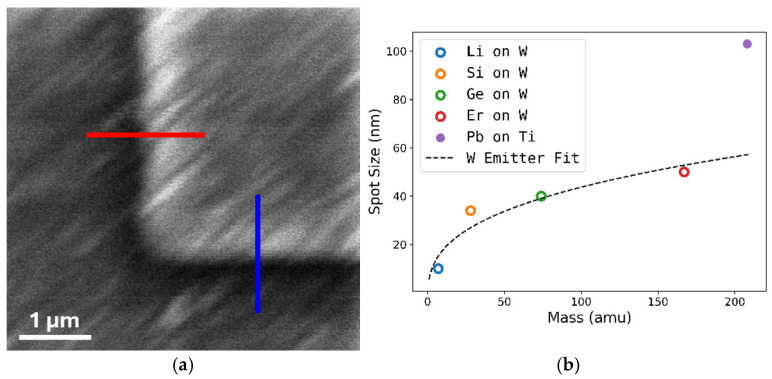
Focusing of Pb on Ti emitter. (**a**) Spot size acquired using 208-Pb^++^ at an acceleration voltage of 80 kV. The red (blue) lines denote the X and Y directions, respectively. (**b**) Comparison of Pb spot size (filled circle) to previously achieved spot sizes on the nanoimplanter using W emitters (open circles). The dashed line is fit to the W emitter values. Adapted from [29] (Li), [5] (Si), [31] (Ge), and [32] (Er).

Using the nI, we measure the Pb^++^ spot size at 80 kV acceleration potential. This is done using secondary electron detection along the edge of an etched Si grid. The step size was 4.88 nm, which is also the pixel size in the generated image. We assume a Gaussian shaped beam, measuring the 80% and 20% intensity pixel distance to extract the full width at half maximum of the Gaussian beam. A picture using 208-Pb^++^ as the imaging ion is shown in Figure 5. The measured spot size is 103 ± 10 nm in X and 104 ± 16 nm in Y, measured along the red and blue linecuts, respectively. For an FIB, this is a relatively large spot size attributed to a combination of multiple effects: (1) the m^1/3^ dependence of the minimum achievable spot size, (2) isotopes with relatively similar abundance partially overlapping in the mass spectrum, and (3) sub-optimal operation of the nI. To understand how far we are deviating from the mass dependence of the expected achievable spot size, we compare our data to previous spot sizes achieved on the nI using W-based emitters. Figure 5b shows that the Pb spot size achieved is much larger than what would be expected from a W emitter. Extrapolating the m^1/3^ dependence, shown as the dashed line, we expect a best spot size of 58 nm. (2) leads to an apparent elongation of the spot due to adjacent isotopes being partially transmitted through the column. By stigmating, we were able to achieve a similar measurement of the X and Y spot size; however, this comes at the cost of overall spot size. Lastly, (3) is due to a leakage current on the objective lens, preventing the application of higher voltages to the objective lens and preventing us from achieving the nominal operating voltage of 100 kV for the nI at which the minimum spot size can be achieved.

## 5. Conclusions

We have performed an exhaustive search of the ASM binary alloy phase diagram database for Pb-containing alloys that are suitable for use as an LMAIS FIB ion source. After identifying eutectic Sn_74_Pb_26_ (at.%) as having a low melting point and combining two ions relevant for fabricating diamond color centers in a single source, we fabricated eutectic Sn_74_Pb_26_ (at.%) sources. The eutectic Sn_74_Pb_26_ (at.%) was found to not wet commercially available W emitters, leading us to fabricate our own emitters out of Ti and Ta wire. The IV curves produced from the emitters show turn-on behavior and stability suitable for operation in a FIB. Running the sources in the FIB we are able to acquire mass spectra showing emissions of Pb^++^, Sn^++^, and Sn^+^ and isotopically resolve these spectra. Further work will explore different coating techniques to enable the fabrication of novel LMAIS sources that do not wet W directly on commercially available W emitters.

## Data Availability

The raw data supporting the conclusions of this article will be made available by the authors on request.
